# Protocol for laboratory rearing and infection tracking of *Rhodnius prolixus* using 3D-printable designs

**DOI:** 10.1016/j.xpro.2025.103894

**Published:** 2025-06-13

**Authors:** Ruby E. Harrison, Ronald Drew Etheridge

**Affiliations:** 1 Center for Tropical and Emerging Global Diseases, University of Georgia, Athens, GA 30602, USA; 2 Department of Cellular Biology, University of Georgia, Athens, GA 30602, USA

**Keywords:** Cell Biology, Microbiology, Special Issue, Protocols in Entomology

## Abstract

Human infections by *Trypanosoma cruzi* propagate via its blood-feeding triatomine vector. Investigating parasite-vector interactions depends upon robust techniques to rear insects and analyze infections. Here, we present a protocol for laboratory rearing and infection tracking of *Rhodnius prolixus.* We describe steps for housing, feeding, and sorting strategies using 3D-printable designs. We also detail procedures for gut dissection, fecal collection, and parasite re-isolation. This protocol describes techniques that support efforts to understand and mitigate vector-mediated Chagas disease transmission.

## Highlights

• Assembly instructions for partially/fully disposable caging for *Rhodnius prolixus*
• Design for artificial blood-feeding system using water balloons and handwarmers
• Guidelines for collection of excreta from *Trypanosoma cruzi*-infected *R. prolixus*
• Procedure to isolate *T. cruzi* from *R. prolixus* gut or excreta for axenic culture

## Abstract (editor highlights)

Publisher’s note: Undertaking any experimental protocol requires adherence to local institutional guidelines for laboratory safety and ethics.

## Graphical abstract

**Figure:**
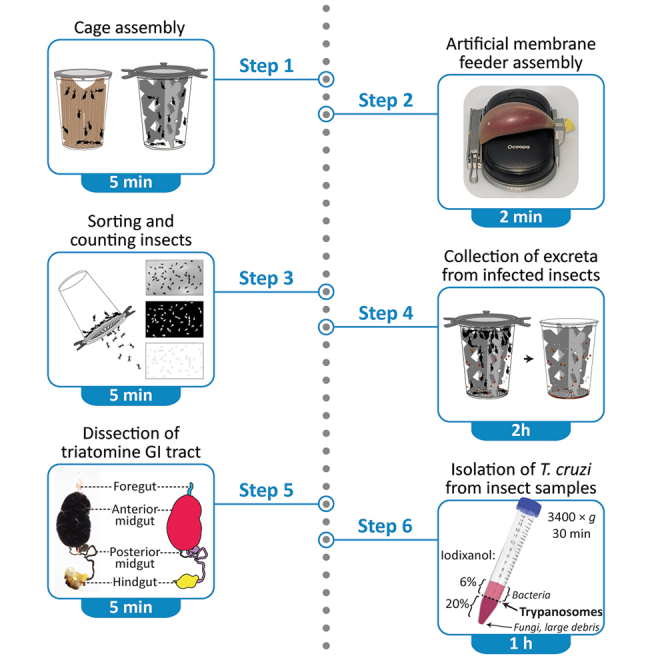

## Before you begin

Insects belonging to the subfamily Triatominae, commonly referred to as triatomines or kissing bugs are primarily hematophagous and can subsist on blood alone throughout the entire duration of their lifespan.^1^ Triatomines require blood meals to progress through successive stages of juvenile development, for subsistence as adults and, in the case of adult females, for egg formation.^2^ This blood-feeding habit underlies the triatomine ability to acquire and transmit the deadly parasite *Trypanosoma cruzi* to vertebrate hosts including humans. Infection with this parasite can lead to Chagas disease, endemic to the Americas, which infects an estimated 6–7 million people and causes approximately 12,000 deaths each year.^3^ Elucidating how *T. cruzi* establishes infection within triatomines is crucial for developing novel strategies to disrupt ongoing transmission in endemic areas.

A barrier for rearing triatomines is provisioning this all-blood diet. To date, no artificial diet has been developed for triatomines, and use of animals for insect colony maintenance requires approval and continual update of animal use protocols, an animal care facility, associated training and reagents for anesthesia, and raises ethical concerns.^4^ Further, triatomines consume large amounts of blood corresponding to volumes as high as 400 μl per individual depending on species and life stage,^5^^,^^6^ which would rapidly lead to exsanguination of small mammals if a large number of insects were allowed to feed. The use of a membrane feeding system with commercially available sources of blood mitigates several of these problems but requires equipment that may not be accessible to all due to economic or other factors. A second major drawback of using triatomines in research is their protracted life cycle, requiring from 4–12 months or even longer to progress from egg to adult depending on species,^7^ environmental conditions including temperature and humidity,^8^^,^^9^ frequency of blood-feeding,^2^ and nutritional quality of blood provided.^6^^,^^10^ Triatomine rearing, therefore, should be as high-throughput and robust as possible to ensure continual availability of high numbers of individuals at the desired life stage.

*Rhodnius prolixus* is a major model organism within insect physiology research^11^ and presents several advantages over other triatomine species including a complete and well-annotated genome^12^ and proven amenability to genetic manipulation using RNAi^13^ and CRISPR-Cas mutagenesis.^14^ Several existing publications detail methods to rear triatomines including *R. prolixus*.^10^^,^^15^^,^^16^^,^^17^^,^^18^^,^^19^^,^^20^ In establishing our own *R. prolixus* colony using these standard protocols, we were confronted with limitations in current husbandry protocols that negatively impacted both colony health and the ability to systematically study *T. cruzi* infection. Recognizing these gaps, we developed updated protocols aimed at making triatomine rearing more efficient and infection studies more robust.

Here, we present a comprehensive toolkit that integrates four key areas: (1) housing, (2) feeding, (3) colony management, and (4) infection tracking. These protocols collectively address the most critical challenges in triatomine husbandry and are designed to be accessible to researchers with minimal prior insect rearing experience. We also introduce new methods to purify trypanosomes from triatomine gut or excreta samples for downstream applications including microscopy, molecular analysis, or placement into axenic culture. Taken together, these form a holistic guide to effectively maintain *R. prolixus* colonies and perform rigorous *T. cruzi* infection studies with the overarching aim of supporting the broader scientific community in efforts to combat Chagas disease.

### Institutional permissions

Before commencing rearing or experimental infection of *R. prolixus*, researchers must ensure they first meet the arthropod containment guidelines defined by their institution. All triatomine rearing and infection assays performed in the development of these protocols were in accordance with ACL-2 (Arthropod Containment Level 2) insectary guidance^21^ established by the University of Georgia Office of Biosafety.

## Key resources table

**Table:** 

REAGENT or RESOURCE	SOURCE	IDENTIFIER
**Bacterial and virus strains**		

*Rhodococcus rhodnii*	American Type Culture Collection (ATCC)	35071

**Biological samples**		

Defibrinated or anticoagulated vertebrate blood; defibrinated sheep blood used in this protocol	E.g., Hemostat Laboratories	Used in this protocol: Hemostat Labs DSBXXX; last three numbers variable and correspond with product volume

**Chemicals, peptides, and recombinant proteins**		

DMEM high glucose, pyruvate-free	GE Lifescience	SH3000303
Fetal bovine serum, non-heat-inactivated	VWR	89510–186
Bovine serum albumin (BSA)	Genesee Scientific	25–529
Iodixanol 60% w/v (OptiPrep)	Serumwerk	1893

**Deposited data**		

Raw data	This paper, File S2	N/A

**Experimental models: Organisms/strains**		

*Rhodnius prolixus* CDC strain	BEI Resources	NR-44077
*Trypanosoma cruzi* Y strain (DTU II)	ATCC	50832

**Software and algorithms**		

PrusaSlicer	Prusa Research	https://www.prusa3d.com/page/prusaslicer_424/
Fiji	Fiji	https://fiji.sc/
Microsoft Excel	Microsoft 365	https://www.microsoft.com/en-us/microsoft-365/excel

**Other**		

32 oz plastic deli containers	Choice	127DM32BULK
Donut lids with snap-on clips	BugDorm	BDC0003_12P https://shop.bugdorm.com/product_info.php?products_id=258
Corrugated cardboard wrap roll	ULINE	S-2714
Guillotine-style paper cutter	Various commercially available	N/A
Circle cutter, ∼7.5 cm diameter	Various commercially available, e.g., American Button Machine	27500 https://www.americanbuttonmachines.com/products/circle-cutter
Medium-fine nylon mesh (small enough weave to prevent 1^st^ instar nymphs from escaping)	Fabric supplier, various	N/A
3D printer	Various commercially available, e.g., Prusa Research	E.g., Original Prusa MK4S 3D Printer kit
Plastic filament, PLA or PETG	Various commercially available	N/A
Plastic filament (PLA) imbued with antimicrobial silver ions	CARBON through Amazon supplier	https://www.amazon.com/Silver-Certified-Anti-Bacterial-Printer-Filament/dp/B0CR8TQDX3/
7.5 cm diameter circular filter paper	VWR	28313-046
Craft pipe cleaners (chenille stems)	Various commercially available	N/A
Water-jacketed glass membrane feeder	ChemGlass	CG-1836-75
Powder-free latex gloves XL	VWR	76319-672
5 cm diameter O-ring	Grainger	29VL75
Tygon tubing	Grainger	S3 E-3603 NSF-51
Hot water bath with circulator pump	Various commercially available	N/A
Autofill water balloons	Various commercially available	E.g., https://www.amazon.com/Bunch-Balloons-Pack-Amazon-Exclusive/dp/B07GW2QQWN
30 mL syringe luer-lock	BD	302832
Syringe dispenser needles	Amazon	https://www.amazon.com/Auniwaig-Plastic-Tapered-Dispenser-Dispensing/dp/B097M65V12?th=1
Electric handwarmer, USB rechargeable	E.g., Ocoopa through Amazon supplier	Temperature setting 37°C–41°C ideal; to fit adapter, must be flat-bottomed, dimensions approximately 8.6 × 4.8 × 1.8 cm. Specific model used in this protocol: https://www.amazon.com/OCOOPA-Rechargeable-Pocket-Sized-Handwarmers-Electronic/dp/B0CC189314/
Featherweight entomology forceps for handling insects	Fisher Scientific	S72110
Carbon dioxide gas cylinder with regulator	Various commercially available	N/A
Sharp forceps for dissection	Fine Science Tools	11251-10
Spring scissors for dissection	Fine Science Tools	91500-09
Disposable scalpel for dissection	VWR	95045-502

## Step-by-step method details

### Preparation and assembly of cages with corrugated cardboard and commercially available lids

**Timing: approximately 1 min to cut cardboard for one cage**

**Figure 1:**
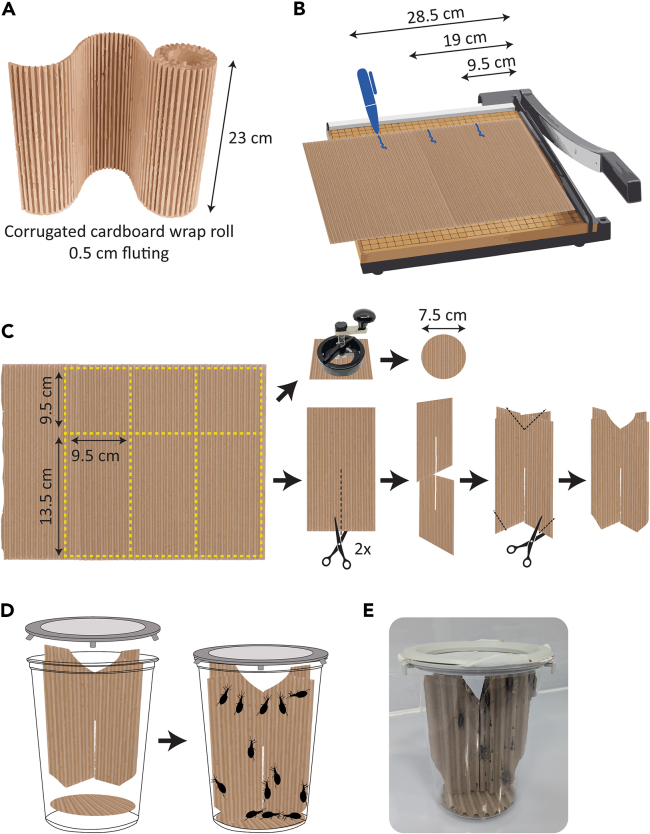
Instructions for high-throughput production of cardboard supports for kissing bug caging (A) Dimensions of corrugated cardboard roll recommended for this method. (B) Diagram indicating how to mark and cut cardboard at 9.5 cm lengths to generate several long strips. (C) Yellow dotted lines show where to cut to produce 9.5 × 13.5 cm rectangles and 9.5 × 9.5 cm squares. Squares are further cut into circles for the cage bottom; rectangles are given central vertical slits to allow slotting of two pieces to make a cross shape and triangles removed from bottom corners and top to allow bugs to more easily access all quadrants of the cage. (D) Diagram showing cage assembly. (E) Photograph of assembled cage with cardboard supports and commercially acquired cage lid.

*R. prolixus* are traditionally housed in reusable plastic containers lined with filter paper to absorb feces and containing vertical cardboard paper supports that serve as refugia and as substrates for oviposition.^20^ Common nuisances of triatomine colonies include bacterial and fungal pathogens that can be vertically transmitted and for which eradication requires continuous extensive sterilization of caging materials.^20^ The protocol described below uses disposable deli cups as cages to avoid persistence of microbial entomopathogens in the insect culture and streamlines cardboard cutting for large-scale, high-throughput preparation. Fluted, corrugated cardboard roll (Figure 1A) is inexpensively acquired in bulk and provides refugia for 1^st^ and 2^nd^ instar nymphs without the need for pleating or cutting multiple holes like the traditional cardboard paper substrate.^20^

1. Cut corrugated cardboard into longitudinal strips: place edge of corrugated cardboard roll flush with guillotine-style paper cutter.
  a. Using the ruler on the paper cutter, mark every 9.5 cm with a pen or marker (Figure 1B).
  b. Cut cardboard where marked to produce several rectangles 9.5 cm × 23 cm.
2. Cut cardboard strips into one rectangle and one square each.
  a. Using the paper cutter and its ruler, cut long rectangles transversely to produce one square 9.5 cm × 9.5 cm and one rectangle 9.5 cm × 13.5 cm (Figure 1C, left).
3. Use a circle cutter or a pair of scissors to produce a circle from the cardboard square approximately 7.5 cm in diameter (Figure 1C, top right).
4. Use a pair of scissors to make a central vertical slit in each cardboard rectangle.
5. Slot two rectangles together to produce vertical “supports” that act as a substrate for insects to climb on.
  a. To improve fit of cardboard supports within the cage (deli cup), clip off the outer bottom corners and a V-shaped indent in the top center (Figure 1C, bottom right).
6. Assemble cage (deli cup) with cardboard circle on the bottom, vertical supports in the middle, and commercially acquired donut lid with medium-fine nylon mesh sandwiched between lower and upper halves on top (Figures 1D and 1E).

**CRITICAL:** Change cages for insects every 2 weeks minimum or whenever excessively soiled; blood-fed triatomines excrete large volumes of urine and feces which dampen cardboard and can eventually lead to mold growth. Deli cup and cardboard pieces can be placed in the freezer for 24 h to ensure any hiding insects or eggs are killed, then disposed of. Donut lids and nylon mesh can be cleaned by soaking in 10% bleach for 2–16 h and rinsed thoroughly before reuse.

### Preparation and assembly of cages with 3D-printed supports and lids

**Timing: dependent on make and model of 3D printer; approximately 3–5 h to print materials needed for one cage**

This cage design is semi-disposable in that deli cup and filter paper are discarded but 3D printed supports and lids are cleaned and reused. 3D printed materials can be generated with regular plastic filament or, optimally, with filament containing silver nanoparticles which has proven antimicrobial^22^ and acaricidal^23^ properties. Silver nanoparticle-containing caging material may therefore potentially help to keep microbial pathogens at bay, as well as mites, which are common deleterious pests of insect laboratory cultures and especially of field-caught triatomines.^24^

**Figure 2:**
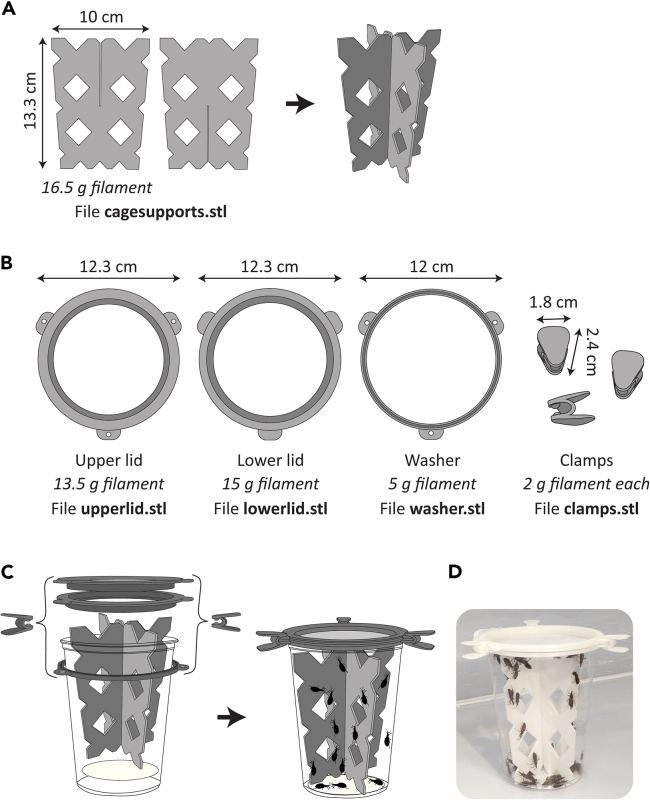
Illustrations of 3D-printed components for insect caging (A) Design of bottom (left) and top (right) flat pieces which are slotted together to form cage supports; dimensions and filament required per item are indicated and are equal for both pieces. (B) Design of cage lid pieces with dimensions and filament needed indicated for each. Recessed areas are colored dark gray. (C) Illustration demonstrating assembly of cage with 3D printed components. Note that nylon mesh cloth is sandwiched between upper and lower lids. (D) Photograph of assembled cage with 3D printed materials.

***Note:*** 3D printed supports have a smooth surface that early-stage juvenile *R. prolixus* may have trouble climbing. Roughening the surface of the supports with coarse-grain sandpaper solves this problem.

7. Design for 3D printed vertical supports and their assembly is presented in Figure 2A.
  a. Printing takes 30 min to 1 h 30 min for one upper and lower support each. Time to print depends on make and model of 3D printer.

8. Design for 3D printed cage lid components is presented in Figure 2B.
  a. Printing takes 1 h 20 min to 2 h 38 min to produce one each of the upper and lower lid pieces, and an additional 30 min to 1 h 30 min to produce the “washer” and clamps.
9. Assemble cage (deli cup) with a 7.5 cm diameter filter paper circle on the bottom, vertical supports in the middle, and 3D printed lid with medium-fine nylon mesh sandwiched between lower and upper halves on top.
  a. To affix the lid, place the washer around the outside of the deli cup under the rim and use three clamps to secure washer and upper and lower lid pieces together (Figures 2C and 2D).

**Table 1:** Suggested rearing densities for *R. prolixus* in 32 oz deli cup caging system

Life stage	Number of individuals per cage
Eggs/1^st^ instar nymphs	No upper limit; minimum of 250
2^nd^ instar nymphs	100–300
3^rd^ instar nymphs	50–100
4^th^ instar nymphs	50
5^th^ instar nymphs	50
Adults	50

*Note:* To obtain high egg yields from adult cages, ensure 18–20 females out of 50 total individuals are present. Adult male and female *R. prolixus* are readily distinguished by a quick visual examination of the terminal abdominal segment (see Sutcliffe *et al.* 2024^20^).

***Note:*** A major advantage of using 3D printed supports is facilitation of maintaining a clean and dry caging environment; a downside is that the design is exposed and offers no refugia to the insects. We have observed no apparent reductions in fitness of our *R. prolixus* housed in these cages compared to traditional housing. However, we recommend careful surveillance for indicators of insect stress potentially arising from exposure following implementation of this caging system.

***Note:*** While *R. prolixus* adult females oviposit on cardboard supports, they do not readily adhere their eggs to the surface of 3D printed supports. This problem is overcome by placing pipe cleaners (crafting style) as a substrate for oviposition inside cages; see further information in “expected outcomes” section and Figures 8B–8D.

**CRITICAL:** Change cages for insects every 2 weeks minimum or when excessively soiled to prevent moist, dirty conditions promoting proliferation of potential pathogenic microorganisms. Deli cup and filter paper can be placed in the freezer for 24 h to ensure any hiding insects or eggs are killed, then disposed of. 3D printed supports, lid pieces, and nylon mesh can be cleaned by soaking in 10% bleach for 2–16 h and rinsed thoroughly before reuse.

***Note:*** While there’s no evident detriment to housing *R. prolixus* stages mixed together, it simplifies husbandry and maintenance considerably to house life stages separately. Suggested densities for each life stage are presented in Table 1 for this caging setup. It’s easiest to handle first instars when blood-fed or to wait until they are second instar nymphs; prior to hatching, transfer eggs retrieved from adult cages by moving pipe cleaner with adhered eggs to a new cage. Allow eggs to hatch and blood-feed nymphs *en masse* before reducing density.

### Assembly of artificial membrane feeding system using water balloons and handwarmers

**Timing: 1 h to produce 3D-printed handwarmer adapter; 1–2 min to fill and assemble a single balloon and handwarmer**

A common device used for triatomine rearing is the glass water-jacketed feeder,^25^ which warms blood to 37°C via attachment to a circulating hot water bath. However, glass feeders are expensive and, because kissing bugs require 1–2 h to feed to repletion, inadequate for efficient feeding of large numbers of cages. The protocol below uses water balloons warmed by electric handwarmers that can be inexpensively scaled up to feed several dozen cages simultaneously, with the added advantage that insects can be fed inside of their incubator or growth chamber.

***Note:*** A video demonstrating balloon filling and attachment to handwarmer is available (Methods video S1).

***Note:*** A comparative table indicating one-time and recurrent costs of caging materials and blood-feeding systems discussed here is presented in Table 2.

**Figure 3:**
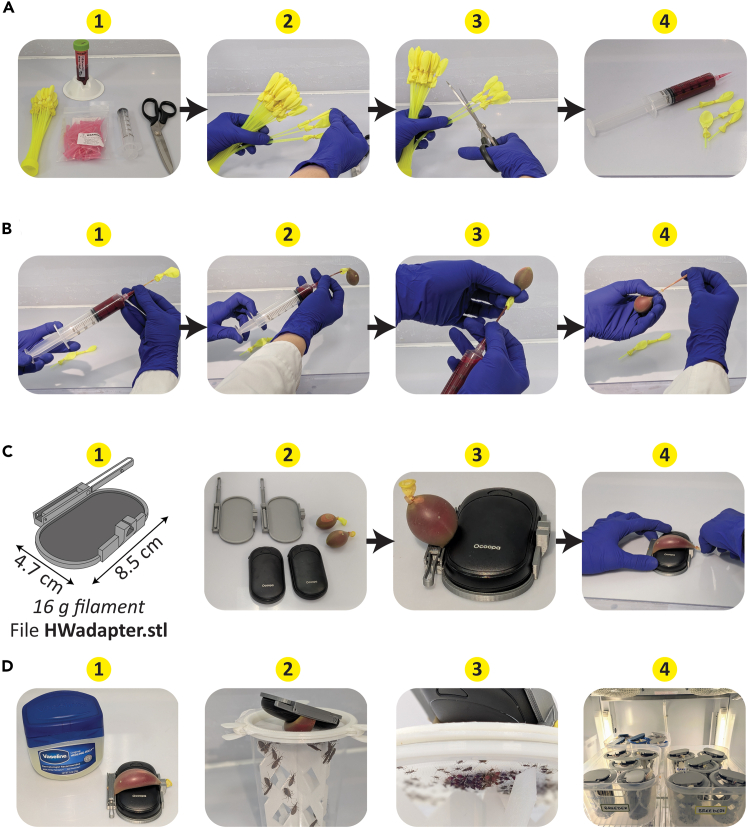
Artificial blood-feeding system using water balloons and handwarmers (A) 1: Materials needed to prepare blood-filled balloons. 2: Manual repositioning of balloons preloaded onto filling straws. 3: Clipping of straws. 4: Prepared blood-filled syringe and balloons. (B) 1: Placement of balloon on syringe using hand to secure straw to pipet tip. 2: Filling of balloon with blood. 3: Removal of blood-filled balloon from straw. 4: Tying knot of filled balloon. (C) 1: Diagram of 3D printed adapter used to secure balloon to handwarmer with dimensions and filament requirement indicated. 2: Photograph of 3D printed adapters, handwarmers, and blood-filled balloons. 3: Photograph of balloon with full end clamped with adapter clasp. 4: Photograph demonstrating securing balloon knot to cavity on adapter distal side. (D) 1: Photograph of handwarmer and 3D printed adapter with balloon secured, and lubricant (Vaseline) used to prevent balloon sticking to nylon netting. 2: Cage of *R. prolixus* adults with overturned artificial feeder atop cage. 3: Underside view photograph showing first instar nymphs gorging from feeder. 4: Photograph of multiple cages feeding from handwarmer + balloon setup within climate-controlled incubator.

***Note:*** Balloons are secured to straws with small rubber bands; do not pull the rubber band all the way off of the straw.

10. Assemble needed materials and make adjustments to water balloons pre-loaded onto straws.
  a. Pull balloons to the end of each straw and trim the straw length (Figure 3A, panels 1–4).

11. Load a syringe with anticoagulated or defibrinated blood.
  a. Place the end of the balloon straw to the syringe tip while holding the straw securely in place with a gloved hand, and gently fill the balloon (Figure 3B panels 1–2).

***Note:*** Volumes of 2.5–10 ml work best; any lower and the latex does not stretch thin enough, while volumes over 10 ml may no longer be heated evenly by the handwarmer.

12. Pinch the end of balloon above the straw and gently pull the filled balloon off of the straw (Figure 3B, panel 3). The rubber band around the end of the balloon will seal it closed.

***Note:*** To ensure no blood leaks during feeding, we strongly recommend additionally tying a knot at the end of the balloon (Figure 3B, panel 4).

13. Place an electric handwarmer in the 3D printed adapter and gently secure the round end of the balloon by pinching the tip of it with the adapter clip, such that the knotted end is free. Then stretch the knot to the adapter hole (Figure 3C, panels 1–4).
14. Turn the handwarmer on and place the feeder upside-down atop the mesh cage lid.
  a. To prevent the balloon from sticking to the cage lid mesh, add a thin coat of Vaseline onto the balloon surface (Figure 3D, panel 1).

**CRITICAL:***R. prolixus* is often found associated in natural settings with its beneficial bacterial endosymbiont *Rhodococcus rhodnii*. *R. rhodnii* has been demonstrated to significantly enhance development, survival, fecundity, and immune function of *R. prolixus*.^26^^,^^27^ Addition of *R. rhodnii* to blood meals at a final concentration of 10^6^ colony-forming units (CFUs) per mL is recommended to support robust *R. prolixus* colony health; methods to prepare bacterial cultures and inoculate blood were recently detailed by Sutcliffe *et al.* (2024).^20^*R. prolixus* acquires *R. rhodnii* in natural settings horizontally from conspecifics via coprophagy.^28^ Hence, routine dietary supplementation with *R. rhodnii* is particularly important when using caging with silver-containing 3D printed supports, which likely kill on contact the bacteria within deposited feces.

***Note:*** Blood from a variety of vertebrate animals is suitable for rearing *R. prolixus,* with pig blood, human blood, and rabbit blood evincing nutritional advantages over bloods from various other animals.^5^^,^^29^ Choice of anticoagulant is also an important consideration, with heparin being preferable to EDTA and or sodium citrate, which negatively impacted insect health.^30^ For routine maintenance of our *R. prolixus* colony and for experimental infections using *T. cruzi,* we utilize sheep blood which has been mechanically defibrinated to prevent clotting.

***Note:*** Adults and juveniles as young as 1^st^ instars readily pierce the latex balloon membrane and feed to repletion (Figure 3D panel 2 shows adults feeding; panel 3 shows bottom view of 1^st^ instar nymphs feeding).

***Note:*** Large numbers of insect stages that consume large volumes of blood (4^th^-5^th^ instars, starved adults) may completely drain a balloon, requiring that a second filled balloon is swapped in to allow all individuals to feed to repletion.

**Table 2:** Costs for equipment needed for *R. prolixus* caging and blood-feeding

Resource	Cost in USD	Initial cost	Recurring cost
**Caging materials for cardboard/commercial lid setup**			

Corrugated Cardboard	$0.15	$4.58 per assembled cage	$0.30 per assembled cage
32 oz deli cup	$0.15		
Donut lid with snap-on clips	$4.28 (reusable)		

**Caging materials for 3D printed supports & lid setup**			

3D printed supports (top & bottom)	$0.99 (reusable)	$3.67 per assembled cage	$0.17 per assembled cage
32 oz deli cup	$0.15		
3D printed lid (upper, lower, washer, & clamps)	$1.29 (reusable)		
Filter paper 7.5 cm diameter∗	$0.02–0.24		

**Traditional glass membrane feeder setup (does not include hot water bath & circulator pump prices)**			

Glass feeder	$233.15 (reusable)	$240.63 per assembled feeder	$0.15 per assembled feeder
XL powder-free latex glove	$0.15		
5 cm o-ring	$7.33 (reusable)		

**Water balloon + handwarmer feeder setup**			

Electric handwarmer	$11.50 (reusable)	$12.21 per assembled feeder	$0.07 per assembled feeder
Autofill water balloon	$0.07		
3D printed handwarmer adapter	$0.48 (reusable)		

*Note:* Costs are for specific items listed in key resources table, for which suppliers and catalog numbers are specified. For 3D printed materials, costs are calculated based on g filament required assuming a typical price of 30.00 USD per kg filament. Cost of 3D printer itself is not included but ranges from 300–1200 USD with price depending on make and model.

∗ Aeropress XL 73 mm coffee filters are a far cheaper alternative to filter paper at as little as $0.015 each when purchased in bulk from some vendors; e.g., pack of 600 from Prudiut, https://www.amazon.com/Replacement-Disposable-Compatible-Aeropress-Espresso-Style/dp/B0DCVZ4WPZ/.

### Facile sorting of kissing bugs on the basis of size and automated enumeration using Fiji software

**Timing: 1.5–3 h to print sieves, 1 min to anesthetize insects, 1 min to sort using sieve, 2 min to process photograph in Fiji and receive tally**

**Figure 4:**
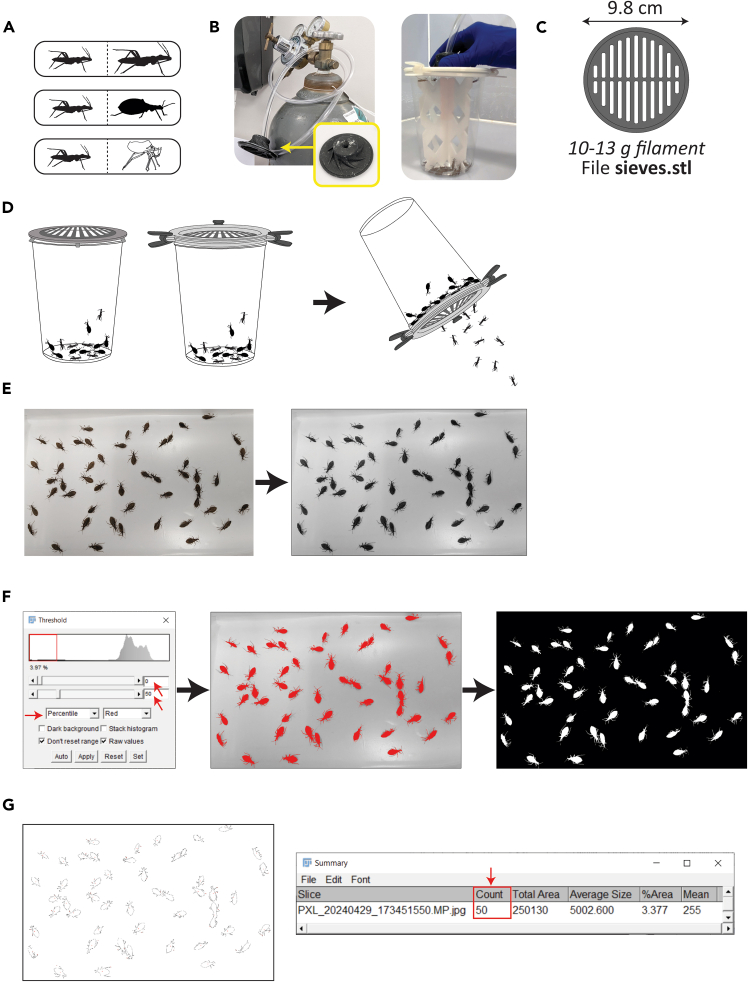
Methods to separate insects on the basis of size and walkthrough of using Fiji software to enumerate individuals (A) Insects of different developmental stages (left), blood-fed versus non-blood-fed (middle), or insects and exuviae or other lightweight debris (bottom) can be separated using a modified sieve. (B) Left: CO_2_ tank with rubber stopper (yellow arrow) used to cover bug cage lid to anesthetize insects. Right: positioning of rubber stopper on cage top to apply CO_2_. (C) Diagram of sieve-style 3D printed cage lid insert with dimensions and required filament indicated. (D) Illustrations of sieve fitted into commercial (left) or 3D printed (center) cage lids in place of nylon mesh and sifting of anesthetized insects (right). (E) Sample photograph of anesthetized bugs on white background before (left) and after (right) conversion to 8-bit file (grayscale). (F) Screenshot of Fiji “Threshold” window with values requiring adjustment indicated by red arrows (left); screenshot of image during threshold adjustment (center); screenshot of image following threshold change application and erosion (right). (G) Screenshot of particle (insect) outlines recognized by Fiji (left) and summary indicating number of particles (insects) recognized (right, red box and red arrow).

Sorting life stages of *R. prolixus* helps in orderly colony maintenance, while separation of blood-fed from non-blood-fed individuals is often helpful for experiments; additionally, separation of insects from debris such as cast-off exuviae following molting helps in maintenance of a clean environment (Figure 4A). Counting individuals rapidly is also helpful when setting up cages of appropriate densities for each life stage (Table 1). This protocol details sorting insects using a 3D printed sieve and automated counting using the cell & particle counter function in the free software Fiji.

15. *R. prolixus* are rapidly and reversibly anesthetized by carbon dioxide.
  a. Fit a properly mounted and secured CO_2_ cylinder with a regulator and Tygon tubing; ensure workspace is adequately ventilated to prevent CO_2_ asphyxiation.
  b. To easily simulate a CO_2_ “pad” similar to those used in *Drosophila* work, add a kitchen sink stopper with a hole punched in the center to the end of the Tygon tubing (Figure 4B, left, yellow arrow; Methods video S1).
16. Cover the mesh of the bug cage top and apply CO_2_ at a gentle flow for one min; anesthetized bugs will drop to the bottom of the cage (Figure 4B, right; see also Methods video S1).
17. Sandwich a 3D printed sieve (Figure 4C) between the upper and lower halves of the lid in place of the nylon mesh.
18. Remove cardboard or plastic supports and filter paper, turn over the cage, and tap the side of the cage gently to allow smaller individuals to go through while retaining larger ones (Figure 4D).
  a. As anesthetized *R. prolixus* will wake up after a few min, be sure to tap insects into a container with upper portion of walls treated with fluon (recently described in detail by Sutcliffe *et al.*^20^) to prevent individuals from escaping.
  b. This method works extremely well to separate insects from exuviae and other lightweight debris during routine cage changes; static electricity causes exuviae to stick to the sides of the plastic deli cup and the insects fall through the sieve.
19. To count anesthetized insects, scatter them so they are fairly well spread out on a white or light-colored container with fluon-treated walls and take a top view picture (Figure 4E, left).
20. Import or transfer the photograph to a computer and open it in Fiji (free to acquire from https://fiji.sc/).
  a. To crop, select the rectangle tool, draw a box around the bugs, and press command+shift+X.
21. From the tool bar ribbon at the top of the program window, click “Image”, then select “Type” and choose “8-bit”. This converts the photo to black and white (Figure 4E, right).
22. Select “Image” again, then “Adjust”, then select “Threshold”.
  a. In this window, select “percentile” from the drop-down menu that is set to “Default” (Figure 4F left panel, left-most red arrow).
  b. Set the top numerical value to 0 (Figure 4F left panel, top right red arrow) and the bottom value to roughly 50 (Figure 4F left panel, bottom right red arrow). Adjust the bottom value up or down as needed to generate a preview image in which the bugs are uniformly highlighted red and where no red is observed in the background (Figure 4F center panel).
  c. Hit “apply”. The picture will convert to a black background with white bug silhouettes (Figure 4F right panel).
23. From top tool bar ribbon, click “Process”, then select “Binary”, and finally “Erode”. This function shrinks the outlines to better separate touching or overlapping individuals.
24. From top tool bar ribbon, click “Analyze” and select “Analyze particles”.
  a. Set “Size (pixel^2^)” to “10 - Infinity”; this prevents counting tiny particles such as debris.
  b. Leave “Circularity” to default “0.00–1.00” setting.
  c. Set “Show” to “Outlines”.
  d. Hit “Apply”. The photo will convert to particles (insects) outlines recognized and quantified (Figure 4G, left). A window labeled “Summary” will pop up, and will indicate the number of particles (insects) counted (Figure 4G, right, red box and arrow).

***Note:*** Sieves with different “pore” (slit) sizes are available in the associated STL file. The sieve with the widest slits is appropriate to separate adults or blood-fed 5^th^ instar nymphs from non-blood-fed 5^th^ instar or younger nymphs. The sieve with medium-wide slits retains blood-fed 4^th^ instar and larger individuals, and the sieve with the narrowest slits retains blood-fed 2^nd^ instar or non-blood-fed 3^rd^ instar and larger nymphs.

Methods video S1. Demonstrations of protocols, related to steps 10–31 Collated clips showing time-lapse footage of balloon + handwarmer feeder setup, anesthetization and sieving of *R. prolixus*, Rutledge feeder assembly, and excreta collection from infected insects. Note that 3D printed stand used to secure Rutledge feeders to cages with rubber bands, while not discussed in the main text, is available to download (rutledgestand.stl).

### Infection with *Trypanosoma cruzi* and subsequent collection of excreta during ensuing blood meals

**Timing: 1 h to decomplement blood for infectious feed; 2 h for infectious feed; 2 weeks following infection to provide a subsequent blood meal; 1 h to collect excreta during subsequent blood meal**

The single-celled parasite *T. cruzi* is a stercorarian, meaning it is transmitted fecally when insects defecate while consuming a new blood meal. These parasites, therefore, reside exclusively within the triatomine gastrointestinal tract. Experimental infection of triatomines and collection of infectious excreta in the laboratory are highly useful for assays in research focused on host-pathogen interactions in this system.

**Figure 5:**
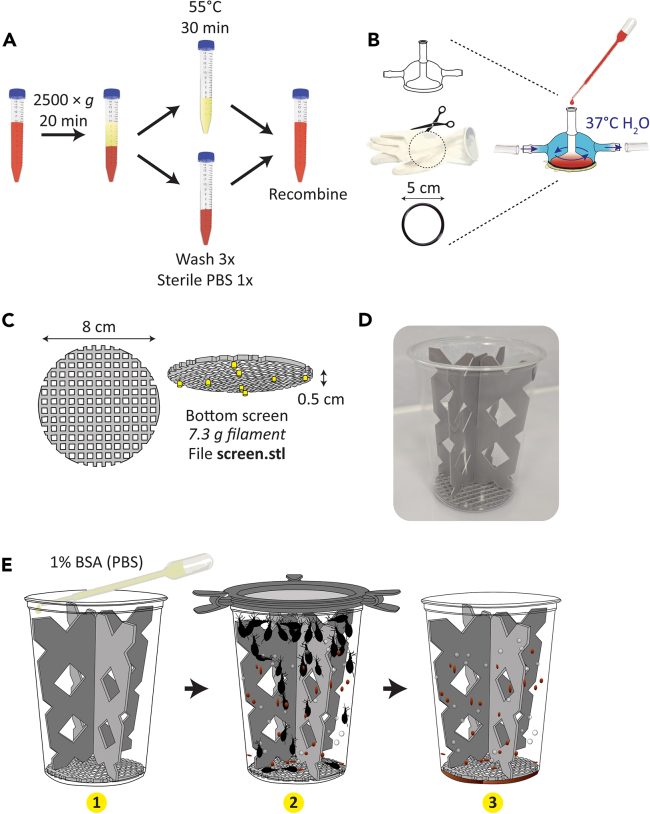
Initial infection of *R. prolixus* with *T. cruzi* and subsequent collection of parasite-containing feces (A) Simple diagram showing work flow to decomplement blood prior to use for experimental infection of *R. prolixus.* (B) Assembly of glass membrane^25^ feeder. (C) Dimensions and filament required to produce 3D printed screen for cage bottom used for excreta collection (left). Screen is elevated off cage bottom by short pegs, colored yellow here to improve visibility (right). (D) Photograph of cage setup for excreta collection. (E) Diagram illustrating work flow to collect excreta. 1: Place screen and plastic (non-antimicrobial) 3D printed supports into deli cup and irrigate supports and cup walls with 10 ml 1% BSA (PBS) buffer to dampen, leaving pool of buffer at cage bottom. 2: Allow insects to blood-feed to repletion. 3: Remove cage lid and insects and gently wash urine/feces off 3D printed materials and cage walls using 1% BSA (PBS). Collect total volume of buffer containing insect excreta from cage and centrifuge at 1000 × *g* for 10 min at 4°C to concentrate into smaller volume.
26. Prepare *T. cruzi* for incorporation into the decomplemented blood.
  ***Note:*** Infection of insects using epimastigote-stage *T. cruzi* is an artificial means of inoculation; in natural settings, triatomines ingest low numbers of blood stream trypomastigote forms from infected vertebrates. However, using epimastigotes for infection is a routine practice in laboratory settings since epimastigotes are extracellular forms that are readily cultivated *in vitro*. Titer of parasites per ml blood used for triatomine infection varies among studies, with commonly used inocula ranging from 5 × 10^6^/ml to 5 × 10^7^/ml.
  a. Pellet the desired number of epimastigotes by centrifugation at 1000 × *g* for 10 min at 4°C.
  b. Aspirate and discard supernatant with a pipette to remove any antibiotics present in the trypanosome culture medium.
  c. Resuspend pelleted parasites directly in decomplemented blood and mix by gently inverting the tube several times.
27. Offer infectious blood to *R. prolixus* using an artificial membrane feeder, allowing insects 2 h to blood-feed (Figure 5B).

***Note:*** Use of a glass membrane^25^ or Hemotek system feeder is preferred for infectious feeds over the balloon & handwarmer system, because the blood is stably maintained at exactly 37°C.

***Note:*** Assembly of a glass membrane^25^ feeder is illustrated in Figure 5B and demonstrated in Methods video S1.

***Note:*** Before excreta collection and examination for trypanosomes, allow at least 2 weeks following experimental infection for parasites to develop and for triatomines to finish digestion of the first blood meal. Small numbers of parasites that are likely to be epimastigotes or spheromastigotes can be observed in excreta from this early time point. Metacyclic trypomastigote forms are not likely to appear in appreciable numbers in the insect excreta until at least 3–4 weeks post-infection.^29^

25. Prior to mixing parasites with blood to feed to triatomines, first decomplement the blood (Figure 5A).
  ***Note:*** This step is required only if using epimastigote-stage *T. cruzi*, the forms of the parasite which proliferate within the insect host*.* Epimastigotes are susceptible to lysis by intact vertebrate complement in whole blood.
  a. Spin whole anticoagulated or defibrinated blood at 2500 × *g* for 20 min at 4°C in a bucket rotor centrifuge to separate packed red blood cells and plasma.
  b. Carefully decant plasma by aspirating with a serological pipet and transfer the plasma to a new tube.
  c. Heat plasma at 55°C for 30 min to inactivate the vertebrate complement-associated proteins.
  d. Cool the plasma on a rotator at 10–20 rpm for 15 min at 21°C–25°C.
  e. While plasma heat-inactivates and cools, resuspend packed RBCs in an equivalent volume of phosphate-buffered saline (PBS) such that the ratio of RBCs to PBS is 1:1 (v/v).
  f. Mix gently by inverting the tube several times.
  g. Centrifuge RBCs suspended in PBS at 2500 × *g* for 10 min at 4°C in a bucket rotor centrifuge.
  h. Using a serological pipet, siphon off and discard the buffer to remove traces of residual plasma, hemoglobin from any lysed RBCs, and the buffy layer containing white blood cells.
  i. Perform this wash (steps 25d-g) three times in total, ending with packed RBCs following removal of the final PBS wash.
  j. Recombine the heat-inactivated, cooled plasma and the washed, packed RBCs at the desired hematocrit (packed cell volume).
    ***Note:*** When decomplementing blood for an infectious feed, the hematocrit (proportional volume of packed RBCs) can be adjusted as desired. One option is to match the natural hematocrit of the host animal; companies supplying whole blood products often provide the packed cell volume of the shipped product. A second common option is to maintain a standardized packed cell volume of 50%, i.e. 10 ml decomplemented blood would be made up of 5 ml washed packed RBCs and 5 ml heat-inactivated plasma.

28. When ready to collect *T. cruzi-*containing excreta from *R. prolixus*, prepare containers prior to placing infected insects within.
  a. Place 3D printed screen (Figure 5C) and vertical supports in the plastic deli cup (Figure 5D).
  b. Irrigate cup walls and supports with 10 ml 1% bovine serum albumin (BSA) in PBS using a serological or transfer pipet (Figure 5E panel 1; Methods video S1).

***Note:*** This buffer moistens all surfaces of the cage so feces do not dry out, which would negatively impact *T. cruzi* viability*.* The protein (BSA) in the buffer keeps epimastigote-stage parasites from sticking to the plastic supports, screen, and deli cup walls.

29. Allow insects to consume blood and defecate freely for 1 h (Figure 5E panel 2; Methods video S1).

***Note:*** For routine maintenance and infection, *R. prolixus* should be allowed to feed for 2 h, but excreta collection should be abbreviated to 1 h to prevent evaporation/drying of feces and urine droplets.

30. Remove the cage lid and carefully transfer the insects to a different container with forceps.
31. Collect the excreta from the container walls and bottom.
  a. Using a serological pipet and working under a laminar flow hood (biological safety cabinet) draw up the buffer pooled at the bottom of the cage and use it to rinse droplets of excreta off the 3D printed supports, screen, and deli cup walls several times (Figure 5E panel 3; Methods video S1).
  b. Following these rinses, draw up the entire volume of buffer containing excreta and transfer to a 15 ml tube.
  c. To concentrate the sample, centrifuge the collected buffer + excreta at 1000 × *g* for 10 min at 4°C and slowly aspirate off the supernatant using a pipette until the desired final volume is reached.

***Note:*** Decontaminate cage materials by soaking in 10% bleach for 2–16 h. 3D printed materials can be reused and deli container autoclaved and discarded.

### Dissection to remove the whole gastrointestinal tract of *R. prolixus*

**Timing: 5–10 min per insect**

**Figure 6:**
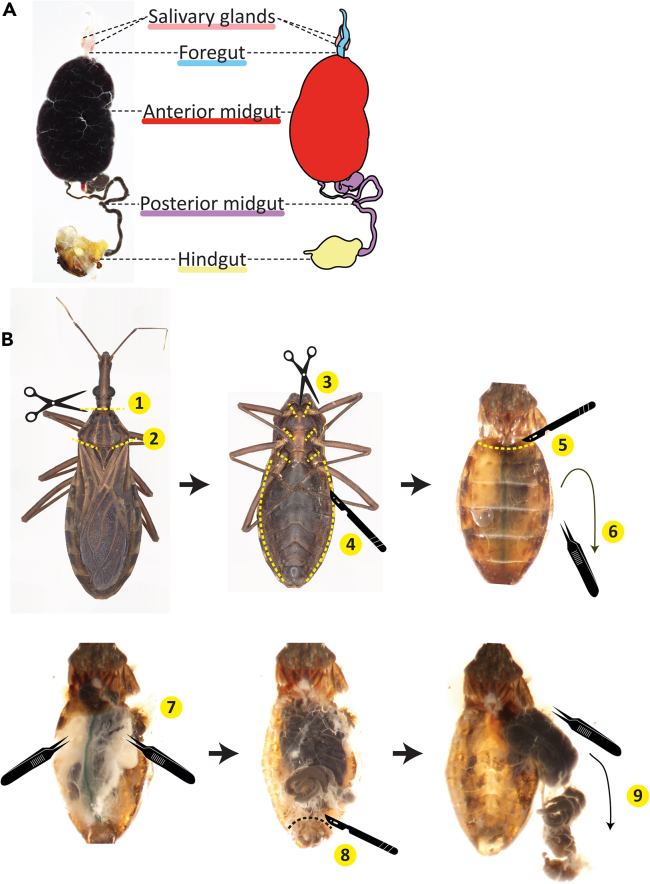
Dissection of *R. prolixus* to remove digestive tract (A) Micrograph (left) and illustration (right) of kissing bug digestive tract regions. Abdominal terminal segment partially obscures hindgut in micrograph. (B) Photographs demonstrating stages of dissection of *R. prolixus* to remove whole GI tract, with steps numbered.

For trypanosome screening, quantification, or isolation, an alternative to collection of excreta is explantation of the digestive tract. The whole GI tract of triatomines is divided into the foregut, which is associated with the salivary glands, the anterior midgut, the posterior midgut, and the hindgut (Figure 6A). *T. cruzi* resides primarily in the hindgut long-term but can also be found in the anterior or posterior midgut regions shortly following its introduction into the insect.

32. Anesthetize insects with CO_2_ application for 3-5 min and/or by keeping on ice or at 4°C prior to dissection.
33. Remove *R. prolixus* head and wings using surgical scissors (Figure 6B #1–2).
34. Flip insect over to view venter and remove legs with scissors (Figure 6B #3).
35. With a disposable scalpel, make lateral slits to separate the dorsal from the ventral abdominal cuticle, avoiding the terminal abdominal segment (Figure 6B #4).
36. Flip insect back over to view dorsum and detach anterior dorsal cuticle edge with scalpel (Figure 6B #5).
37. Using needle-nosed fine forceps, slowly peel the upper piece of cuticle down (towards the posterior) and gently detach from the terminal abdominal segment by tearing off (Figure 6B #6).
38. Remove the heart (green vertical line) and fat body (white tissue) from the dorsal side of the body cavity using forceps (Figure 6B #7).
39. Detach the terminal abdominal segment from the remainder of ventral cuticle with a scalpel, making a semi-circular cut anterior to it (Figure 6B #8). The cut should only be of the ventral cuticle itself, such that the scalpel cutting edge is positioned underneath the exposed GI tract and does not rupture it.
40. Carefully explant the entire digestive tract with the abdominal terminal segment still attached to the hindgut by pulling it in a posterior direction out of the thorax (Figure 6B #9).

***Note:*** The hindgut is closely associated with the terminal abdominal segment and most efforts to isolate it result in its puncture. With exceptional care it is possible to dissociate the two tissues and is more easily done in adult females than in males. It is fine to leave the hindgut and terminal abdominal segment stuck together prior to tissue maceration using a bead homogenizer or pestle when checking for *T. cruzi*.

### Isolation of *T. cruzi* from insect excreta or gut homogenate

**Timing: 45 min to 1 h**

Presence of *T. cruzi* in regions of the triatomine gut informs the success and kinetics of infection, while presence of infectious stage *T. cruzi* in excreta indicates parasite transmission potential. While *T. cruzi* quantification can be accomplished by molecular methods such as qPCR, visual assessment is required to distinguish the non-infectious replicating epimastigotes from the infectious non-dividing metacyclic trypomastigotes. This is vastly facilitated by separation of trypanosomes from other debris in the excreta or gut lumen. Of greater interest still is the potential to rapidly and easily isolate *T. cruzi* from wild triatomines for long-term axenic culture, permitting in-depth genetic analysis and further laboratory experimentation. Cultivation of trypanosomes from insect extracts is a constant battle against contaminating microorganisms, particularly fungi.^31^^,^^32^ This protocol uses a simple two-layer gradient column to separate trypanosomes from all fungi, all visually obstructing debris, and most or all bacteria in a sample. Validation of this protocol is presented below in the “expected outcomes” section.

41. Prepare sterile solutions of 6% Optiprep (iodixanol) and 20% Optiprep, using as the diluent Dulbecco’s Modified Eagle’s Medium (DMEM) containing 15% heat-inactivated fetal bovine serum (FBS).

***Note:*** Store solutions at 4°C and keep on ice while preparing the density gradient column.

***Note:*** Culture media other than DMEM such as liver infusion tryptose (LIT) or brain-heart infusion (BHI) are also suitable diluents.

**CRITICAL:** Ensure diluent contains 10%–15% serum; protein prevents epimastigote-stage *T. cruzi* from sticking to the sides of the tube and failing to migrate though the column.

**Figure 7:**
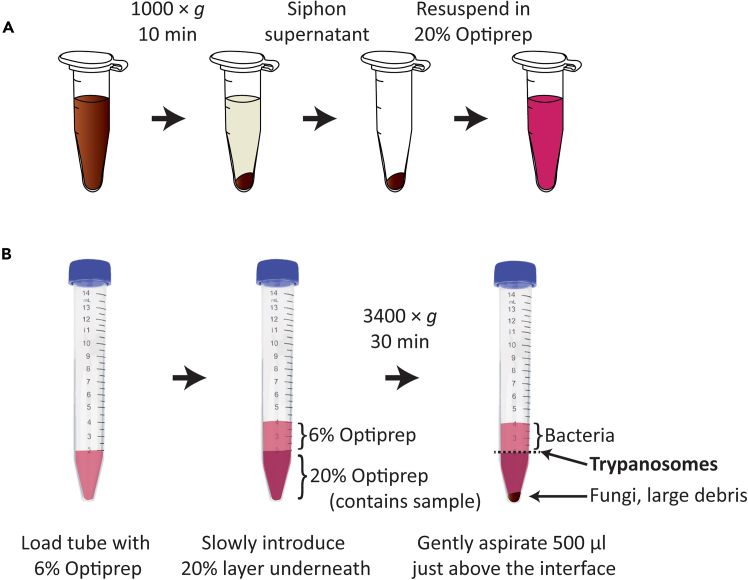
Isolation of pure trypanosomes from *R. prolixus* excreta or gut homogenate (A) Diagram of steps to prepare trypanosome-containing sample for loading onto gradient column. (B) Illustration of work flow to make and centrifuge two-layer gradient, and positions of fungi, bacteria, and trypanosomes following centrifugation.
43. Add 2 mL of 6% Optiprep to a 15 mL conical and keep chilled on ice (Figure 7B, left).
44. With a pipet or a syringe, slowly add the sample (2 ml, 20% Optiprep) to the 15 ml conical, adding it under the initial 6% layer (Figure 7B, center).

***Note:*** Gradually move the pipet or syringe tip upwards when adding the sample/20% layer to the column, such that the tip is continually hovering just underneath the 20%–6% interface.

42. Prepare sample to load onto column (Figure 7A):
  a. Pellet all material including trypanosomes in excreta or gut homogenate sample by centrifugation at 1000 × *g* for 10 min at 4°C.
    i. To prepare *R. prolixus* gut homogenate, place either the dissected whole GI tract or just the hindgut (where trypanosomes are densest) into a 1.5 mL microcentrifuge tube containing 400 μl sterile phosphate-buffered saline (PBS) and macerate using a sterile pestle or beads in conjunction with a bead homogenizer.
  b. Using a pipette, siphon off and discard supernatant.
  c. Resuspend pellet in 2 mL of 20% Optiprep and place on ice.

**CRITICAL:** Take care to avoid mixing the two layers. Keeping all materials as cold as possible and working slowly helps prevent the layers from mixing together.

45. In a pre-chilled bucket rotor centrifuge, spin the 15 mL conical tube containing the two-layer gradient column at 3400 × *g* for 30 min at 4°C.

***Note:*** Set the centrifuge deceleration power to low or zero - this will extend the spin time but prevents the layers from mixing during centrifuge braking.

***Note:*** Centrifugation induces fungi and large debris to form a pellet at the bottom of the tube, causes trypanosomes to migrate to the interface of the 6%–20% layers, and pushes most bacteria above the interface within the 6% layer (Figure 7B).

46. With a pipet, slowly draw up 500 μl of the 6% layer immediately above the interface, moving the pipet tip slowly in a circular fashion to aspirate trypanosomes from as much of the interface surface area as possible (Figure 7B, right).
47. Transfer 500 μl containing trypanosomes to a new sterile tube and use for microscopy, cultivation, or as otherwise desired.

***Note:*** It is helpful to practice this method once or twice with pure trypanosomes obtained from an *in vitro* culture. Very high numbers of trypanosomes (e.g., over 10^7^) will be visible to the naked eye at the 6%–20% interface as a white layer. To better visualize the interface between the 20% and 6% layers, back-light the tube by positioning it in front of a window or other light source.

## Expected outcomes

We adapted the use of disposable deli cups and either fluted cardboard or 3D printed materials as cages to make *R. prolixus* rearing more efficient and, most importantly, as a strategy that may help to counteract pathogens that are deleterious to colony health. Bacteria, fungi, and mites that are pathogenic/parasitic or opportunistic are common nuisances in triatomine cultures.^20^ Our two styles of caging, when implemented as instructed with frequent changes, help to keep conditions clean and prevent the accumulation of excess moisture which favors proliferation of microbes and mites. Both styles pose advantages and disadvantages: fully disposable caging with cardboard supports negates the need for washing and reusing components, but requires labor up front to cut the cardboard pieces, while 3D-printed supports are rapidly assembled but must be cleaned between uses. Wide varieties of plastic filament used for 3D printing are commercially available, including filament imbued with silver nanoparticles. Silver nanoparticles are demonstrated to have potent antibacterial, antifungal, and miticidal properties when incorporated into a variety of materials^23^^,^^33^ and we propose 3D-printed silver-imbued caging components are likely to kill these pathogens on contact.

**Figure 8:**
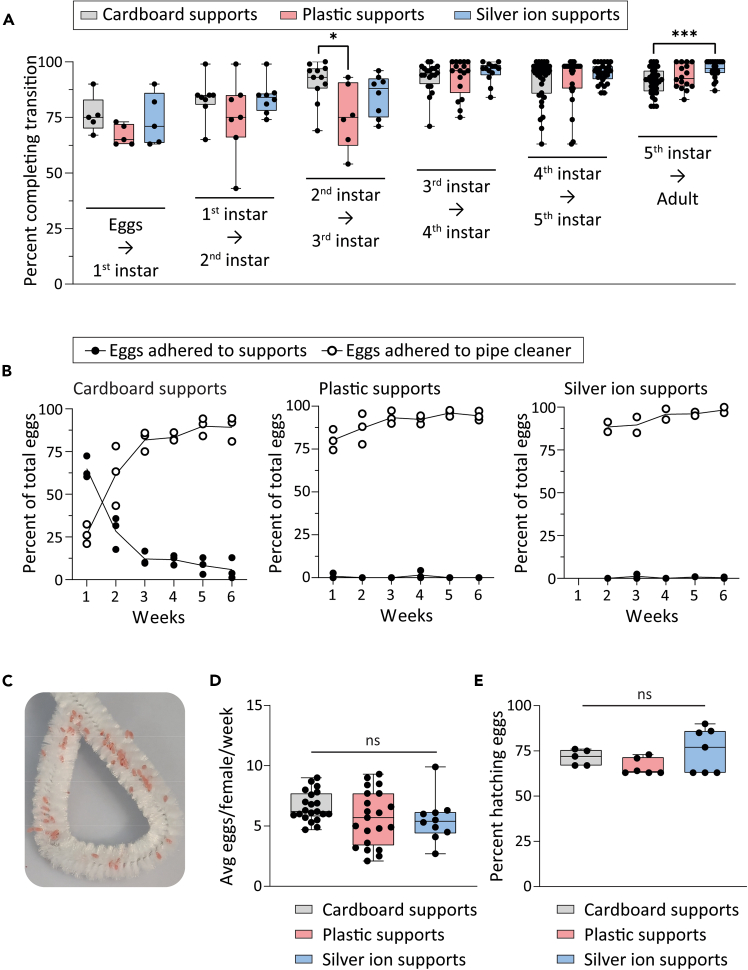
Development, fecundity, and fertility of *R. prolixus* housed in disposable caging (A) Percentage of individuals to progress (molt) from each developmental stage to the next. Dots represent cohorts containing 50-150 individuals. Significant differences among treatments determined by one-way ANOVA are indicated with asterisks ∗ p < 0.05, ∗∗ p < 0.01, ∗∗∗ p < 0.001. (B) Percentages of eggs found adhered to supports or to pipe cleaner out of total for cages using cardboard (left), plastic (center), or silver ion-containing (right) supports over the course of six weeks. Points represent mean values for 2 (silver ion supports) or 3 (cardboard, plastic supports) replicate cages containing 50 adults each. Negligible percentages of eggs were adhered to cage lid mesh or fell to the cage bottom; these data are not shown here but are can be viewed in File S2. (C) Photograph of *R. prolixus* eggs adhered to pipe cleaner. (D) Box-and-whiskers plots indicating mean and quartile values for eggs laid per female *R. prolixus* per week. Dots represent values for replicate cages containing 50 adults of which 19-35 were females. No significant differences among treatments were detected by one-way ANOVA. (E) Proportions of eggs hatching within 2 weeks post-oviposition. Dots represent replicates consisting of 250-500 eggs each laid on pipe cleaner. No significant differences among treatments were detected by one-way ANOVA.

We compared developmental progression of *R. prolixus* reared from egg to adulthood using cardboard, 3D printed plastic, or 3D printed silver nanoparticle (silver ion) cage supports. All types of caging supported normal development with minimal loss; additionally, supports made from silver-containing filament significantly increased the proportion of 5^th^ instar nymphs to progress to adulthood (Figure 8A). One possible reason for this is that keeping environmental microorganisms to a minimum kept pathogenic bacteria or fungi at bay, improving the number of 5^th^ instars to successfully complete the final step in development. Based on our observations of the percent individuals successfully progressing from each life stage to the next and the average time required, we formulated a “Colony Calculator” spreadsheet in Excel that automatically estimates the expansion of each life stage of a colony over time. We have also included a convenient calculator that can help researchers determine the number of breeding adults and time required to generate a specified number of age-matched individuals in order to plan for future experiments (File S1). These calculations are formulated for *R. prolixus* maintained in standardized rearing conditions in a controlled environmental chamber kept at 28°C, 70% relative humidity, with a 12 h:12 h light/dark cycle and with blood meals (defibrinated sheep blood) offered to all life stages once per week.

Adult cages with 3D printed supports lack highly textured surfaces preferred by *R. prolixus* for oviposition. This is easily overcome by provisioning cages with crafting pipe cleaners. *R. prolixus* housed in caging with cardboard supports actually demonstrate a preference for oviposition on pipe cleaners versus cardboard and change their oviposition behavior over time accordingly (Figure 8B). Pipe cleaners with adhered eggs (Figure 8C) are easily removed from adult cages on a weekly basis and placed in new caging prior to hatching of first instar nymphs. We found that *R. prolixus* housed in either cardboard, plastic, or silver ion imbued supports laid equivalent numbers of eggs on pipe cleaners (Figure 8D) and that these eggs showed no differences among treatments in their propensity to hatch (Figure 8E).

**Figure 9:**
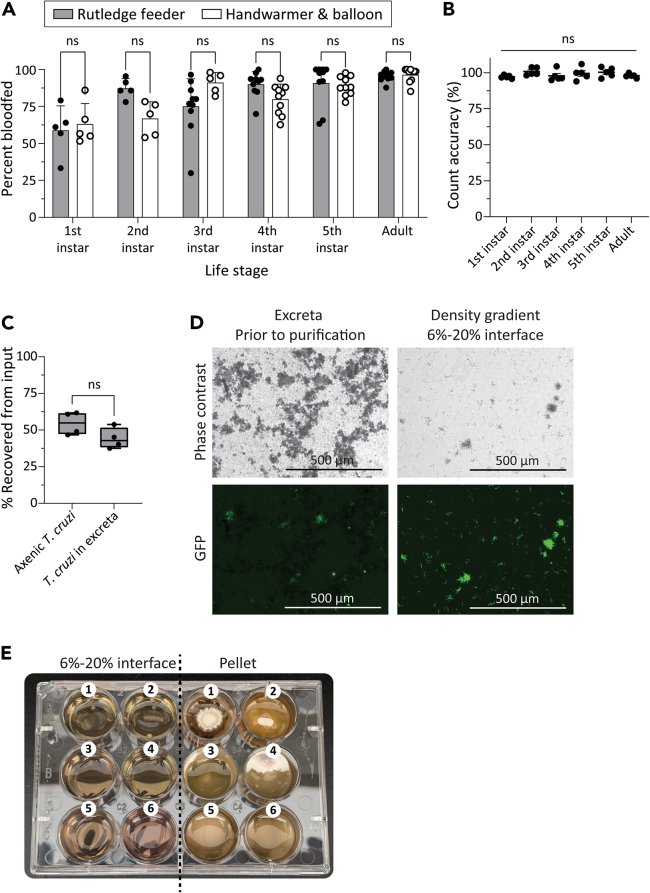
Efficiency of artificial feeders, automated counting, and density gradient isolation of *T. cruzi* (A) Percentages of individuals feeding to repletion on blood offered from glass membrane^25^ or handwarmer & balloon feeders. Dots indicate percent fed individuals for cohorts of 100–300 (1sts and 2nds) or 40–60 (3rds - Adults), with 5–10 replicates per treatment and life stage. Pairwise t-tests indicated no significant (ns) differences between treatments for each life stage. (B) Accuracy of automated insect enumeration by Fiji, represented as percent of true count. Dots represent percent accuracy for images of 100-300 (1st and 2nd instar) or 50–100 (3rd instar - Adult) individuals, with five replicates for each life stage. No significant differences in Fiji count accuracy among life stages were detected using one-way ANOVA. (C) Percent parasites recovered from 500 μl aliquot of 6%–20% interface of density gradient column following purification of a sample with a known starting number of epimastigote-stage *T. cruzi*. Recovery of parasites from axenic culture in LIT medium was not significantly different than recovery of parasites from *R. prolixus* excreta samples by Student’s t-test. Bar-and-whisker plots indicate mean and quartile values, while dots indicate values for 4 replicates per treatment. (D) Representative micrographs of a sample containing GFP-expressing *T. cruzi* in a collected excreta sample prior to purification (left), compared to the 6%–20% interface fraction following density gradient centrifugation (right). (E) Representative image of density gradient column fractions following 7 days’ culture in LIT medium at 28°C. Interface fractions evince no non-trypanosome microbial growth whereas pellet fractions following column centrifugation show significant growth of bacteria and hyphal fungi. Six replicates are numbered; i.e., the cultured interface and pellet fractions for sample #1 correspond to plate wells A1 and A3, respectively.

We modified an electric handwarmer-based feeding system previously developed for mosquitoes^34^ to supply the larger volumes of blood required by triatomines by using water balloons as artificial membranes. All life stages of *R. prolixus* exhibited no significant differences in blood-feeding from the balloon + handwarmer apparatus compared to traditional glass water-jacketed feeders (Figure 9A). Automated counting of anesthetized insects using Fiji resulted in counts ranging from 95% to 104% accuracy with a tendency to under- rather than over-predict the true number, and with reduced precision when tallying 3^rd^–5^th^ instar nymphs, which vary in size more than other life stages when comparing blood-fed and non-blood-fed individuals (Figure 9B).

Methods to conduct triatomine infection assays using *T. cruzi* are described in detail elsewhere^35^ and are briefly recapitulated here to optimally illustrate the full work flow of infection from beginning to end. We present detailed instructions to remove and identify the major compartments of the triatomine GI tract, which have not previously been published to our knowledge, and a novel setup again relying on 3D-printed caging structures that allows collection of insect excreta.

A key protocol presented in this collection that may significantly aid triatomine-trypanosome research however is the reisolation of *T. cruzi* from insect gut homogenate or excreta using density gradient column purification. Gradient column isolation has become a standard method to isolate sporozoite-stage *Plasmodium* parasites from mosquito salivary glands^36^ but has not previously been applied to isolation of trypanosomes from insect hosts. Current methods involve direct cultivation of insect gut extracts in medium containing potent antibiotics and antimycotics, sometimes with repeated dilution or transfer of visible parasites to new media to “outrun” contaminating microorganisms or in conjunction with use of U- or V-shaped tubes that aim to separate microorganisms on the basis of motility.^32^ The chief difficulty lies in eliminating fungal contaminants, which differ in their susceptibility to antimycotics; further compounding this problem, trypanosomes themselves are highly susceptible to certain antimycotics.^37^ We found that epimastigote-stage *T. cruzi* migrate to the interface of a density gradient with two layers consisting of 6% and 20% iodixanol. 37%–62% of trypanosomes loaded onto the column are recaptured at the interface (Figure 9C). Recovery is slightly lower for parasites in excreta compared to in axenic culture, likely owing to clustering of epimastigotes around fecal debris that causes them to migrate to the bottom of the column (Figure 9C). While some bacteria co-migrate to the interface alongside *T. cruzi*, density gradient purification removes all debris, vastly facilitating microscopic examination and life stage classification of parasites (Figure 9D). We have consistently found that fungi migrate to the very bottom of the column following centrifugation (Figure 9E).

In our experiments, transfer of *T. cruzi* collected from the column interface into LIT medium containing penicillin at 100 U/ml and streptomycin at 100 μg/ml is sufficient to rapidly eliminate remaining contaminating bacteria. An exciting prospect of this method is its potential for facile, high-throughput isolation of circulating *T. cruzi* strains for genome sequencing and/or functional analysis from triatomines in regions with a high incidence of Chagas disease. Fewer logistical barriers exist to trap insects compared to mammalian hosts, and *T. cruzi* numbers range from 10^4^–10^6^ in insects compared to <10^4^ in mammals with acute phase infection or near-undetectable numbers in mammals with chronic phase infection. To test the rigor of our method, we have successfully reisolated and achieved axenic culture of *T. cruzi* from *R. prolixus* we experimentally infected 41/41 times (100% success rate).

## Quantification and statistical analysis

Percentages of individuals to progress from one developmental stage to the next, numbers of eggs laid, and proportions of hatching eggs, and comparison of Fiji count accuracy for different life stages were compared among treatments using one-way ANOVA following initial tests to confirm a) homogeneity of variances by Bartlett’s test and b) normal distribution of residuals by Shapiro-Wilk test. Proportions of insects that fed on glass versus balloon + handwarmer artificial feeders presented in Figure 9 were compared using pairwise Mann-Whitney t-tests, using the two-stage step-up method of false discovery rate. Recovery of *T. cruzi* following density gradient purification was compared between treatments by Student’s T-test. Statistical analysis and generation of figures was performed using GraphPad Prism v. 10.3.1. Figures were assembled and annotated using Adobe Illustrator. Methods video S1 was compiled and annotated using Adobe Premier Pro. TinkerCad (https://www.tinkercad.com/) was used to design all 3D printed structures.

## Limitations

These protocols are optimized for the *R. prolixus* CDC strain and may not work as well for other *R. prolixus* strains or for different triatomine species. Appropriate densities of insects in 32 oz plastic cups and estimated development times in particular will vary highly among triatomine genera and species. Only select *Rhodnius* species adhere their eggs to a surface, so use of pipe cleaners as oviposition substrates won’t work for many other triatomines. Larger triatomine species such as *Triatoma* sp. drink larger volumes of blood than *Rhodnius* sp., so the balloon/handwarmer artificial feeding system may need to be scaled up to accommodate this. Similarly, sieves designed for sorting *R. prolixus* life stages will need to be resized for different-sized species. The “Colony Calculator” spreadsheet (File S1) is customized for *R. prolixus*, and will need adjustment to be applicable to other triatomine species, many of which have longer life spans than *Rhodnius* sp*.* Finally, while we observed no deleterious effects of silver nanoparticle-containing plastics on *R. prolixus*, it is important to note that silver nanoparticles have acaricidal activity via disruption of mite and tick exoskeletons; hence, care should be taken when first introducing these materials to ensure triatomines, especially early stages, are not also harmed by contact with silver.

Regarding our method to isolate *T. cruzi* from infected triatomines, an important limitation to consider is that the density gradient purification of *T. cruzi* was optimized using experimentally infected laboratory-reared *R. prolixus*. Lab-reared triatomines harbor gut microbial communities that are significantly reduced in complexity compared to those found in natural settings.^38^ Insects collected from the field additionally often harbor antibiotic-resistant bacteria.^39^ Hence, isolation of trypanosomes from field-caught triatomines for the purpose of placement into axenic culture may have reduced rates of success compared to those reported here.

## Troubleshooting

### Problem 1

*R. prolixus* show increased mortality; insect corpses are black or very dark, soft, and bloated. 5^th^ instar nymphs 2-3 weeks post-blood meal may show highest losses (Protocol steps 1-9).

### Potential solution

We periodically see this in our culture and informally call it “the black death”. It often occurs when insects have been exposed to pathogenic bacteria, e.g. following contamination of blood used for feeding. We also hypothesize it may occur when insects have been kept in conditions that induce commensal bacteria in the digestive tract to over-proliferate. To solve this problem, 1) ensure caging is changed frequently and is not over-saturated with excreta; 2) streak new plates of *R. rhodnii* to ensure symbiotic bacterial stocks have not been contaminated; 3) verify sterility of blood used for feeding by culturing in antibiotic-free media or performing PCR to detect bacterial DNA^40^; and 4) thoroughly clean and sterilize materials reused in rearing including glass feeders, cage lids, and 3D printed supports if in use.

### Problem 2

Water balloons used in artificial feeding setup leak or burst (Protocol steps 10–14).

### Potential solution

There are an immense number of vendors of autofill-style water balloons online, and quality of the product is variable. Latex degrades over time, especially following exposure to light, air, and heat. Ensure balloons arrive in good condition and store them in a sealed container (e.g., plastic bag with air removed) in the dark (e.g., box or drawer) to prolong their lifespan.

### Problem 3

Automated counting of individuals using Fiji shows significant inaccuracy (Protocol steps 20–24).

### Potential solution

First, ensure insects are as well separated from each other as possible when taking pictures used for counting. Overlapping or clumped insects are not easily detected as individuals by Fiji. Second, the “erode” function can be used repeatedly to shrink insect outlines to reduce incidence of overlap.

### Problem 4

During excreta collection, infected insects do not feed and defecate freely, or excreta from a single individual is desired rather than from a large cohort (Protocol step 29).

### Potential solution

Ensure blood is fresh (<2 weeks old) and contains *R. rhodnii*, which acts as a phagostimulant for *R. prolixus*. Insects that molted recently (<1 week) often do not blood-feed; offering blood 2 weeks following molting usually results in high gorging rates. Adult *R. prolixus* that have been starved for extended periods of time (6+ weeks) also show reduced feeding, so avoid over-starving individuals.

In the 32 oz plastic container setup, low densities of individuals (e.g. <20 4ths-Adults, <100 1sts-3rds) typically blood-feed poorly. If excreta from few or a single individual is desired, one solution is to add additional uninfected bugs to the cage to boost overall gorging and subsequent defecation. A second solution is to reduce the container volume so vertical distance to the feeder is reduced. 8 or 12 oz containers with the same diameter opening (i.e., for which the same lids can be used) are commercially available and require only shortening of the internal supports. We have found individual adult *R. prolixus* from our colony will blood-feed and defecate in 8 oz containers, while they will not do so in 32 oz containers. However, if the total number of parasites is low in the excreta from a single individual, note that it may be difficult to recover appreciable numbers of trypanosomes from the density gradient interface since this method incurs 50%–60% loss of the input.

### Problem 5

Density gradient purification of *T. cruzi* results in lower recovery of parasites at interface than expected (Protocol steps 41–47).

### Potential solution

Any culture medium or buffer can be mixed with iodixanol to form the gradient, but if the media is low in protein, epimastigote-stage parasites will stick to the sides of the conical or test tube. Ensure the media or buffer contains serum or a solubilized protein such as BSA at a minimum of 1% to prevent this.

### Problem 6

Fungi or significant amounts of bacteria migrate to the density gradient 6%–20% interface (Protocol steps 41–47).

### Potential solution

A limitation of our *T. cruzi* purification protocol discussed above is its optimization for our *R. prolixus* colony, which has a gut microbiota of low complexity and which includes few fungi. Potential solutions to better separate *T. cruzi* from contaminating microorganisms in more complex samples are A) to increase centrifugation time from 30 min to 45 or 60 min, and/or B) to introduce additional layers to the gradient. Pilot experiments we conducted with three layers consisting of 30%/15%/6% showed the majority of trypanosomes migrated to the 6%–15% interface and improved separation from bacteria, but resulted in lower recovery than a simple two-layer column. If lower recovery of parasites is an acceptable trade-off for a cleaner sample, test a three or four-layer density gradient.

## Resource availability

### Lead contact

Further information and requests for resources and reagents should be directed to and will be fulfilled by the lead contact, R. Drew Etheridge (ronald.etheridge@uga.edu).

### Technical contact

Technical questions on executing this protocol should be directed to and will be answered by the technical contact, Ruby E. Harrison (ruby.harrison25@uga.edu).

### Materials availability

We will gladly provide trial samples of 3D printed materials upon request. All 3D print designs are available to download as STL files associated with this manuscript.

### Data and code availability

All raw data used in this manuscript are available to download (File S2).

## Acknowledgments

This work was supported by the National Institutes of Health (R01AI163140 and R01GM144545 to R.D.E.; T32AI060546 to R.E.H.). Jena A. Johnson provided training for the VHX Keyence Imaging System, a shared resource of the UGA Department of Entomology, used for images of *R. prolixus* and its digestive tract. The clip portion of the handwarmer adapter was modified from original dialysis-style clip found at https://www.printables.com/model/6345-dialysis-clamp-laboratory. Justin Wiedeman provided helpful suggestions adapting Fiji to counting anesthetized insects. Kevin J. Vogel provided adult *R. prolixus* and initial training on CDC official rearing methods^20^ used as foundations to start initial colony maintenance in the Etheridge lab.

## Author contributions

R.E.H. and R.D.E. conceived protocols and designed 3D-printed structures. R.E.H. collected and analyzed the data and created figures and tables. R.E.H. and R.D.E. wrote and revised the manuscript.

## Declaration of interests

The authors declare no competing interests.

## Supplemental information

File S1. “Colony Calculator” spreadsheet (Excel), related to expected outcomes See “tutorial” comment boxes on spreadsheet tab 1 to navigate how to use this tool

File S2. Raw data used in this manuscript, related to resource availability

File S3. STL files used in this protocol, related to step-by-step method details
